# CircMAPK9 promotes the progression of fibroblast-like synoviocytes in rheumatoid arthritis via the miR-140-3p/PPM1A axis

**DOI:** 10.1186/s13018-021-02550-y

**Published:** 2021-06-21

**Authors:** Zhihuan Luo, Shaojian Chen, Xiaguang Chen

**Affiliations:** Department of Sports Medical, The Affiliated Ganzhou Hospital of Nanchang University, Ganzhou People’s Hospital, No.17 Hongqi Avenue, Zhanggong District, Ganzhou City, 341000 Jiangxi Province China

**Keywords:** Rheumatoid arthritis, Fibroblast-like synoviocytes, circMAPK9, miR-140-3p, PPM1A

## Abstract

**Background:**

Rheumatoid arthritis (RA) is a chronic inflammatory joint disease, and fibroblast-like synoviocytes (FLSs) are key effector cells in RA development. Mounting evidence indicates that circular RNAs (circRNAs) participate in the occurrence and development of RA. However, the precise mechanism of circRNA mitogen-activated protein kinase (circMAPK9) in the cell processes of FLSs has not been reported.

**Methods:**

The expression levels of circMAPK9, microRNA-140-3p (miR-140-3p), and protein phosphatase magnesium-dependent 1A (PPM1A) were determined by quantitative real-time polymerase chain reaction (qRT-PCR) or western blot assay. Cell proliferation was examined by 3-(4,5-dimethylthiazol-2-yl)-2,5-diphenyltetrazolium bromide (MTT) assay. Cell apoptosis and cycle distribution were assessed by flow cytometry. Cell migration and invasion were tested by transwell assay. All the proteins were inspected by western blot assay. Inflammatory response was evaluated by enzyme-linked immunosorbent assay (ELISA). The interaction between miR-140-3p and circMAPK9 or PPM1A was verified by dual-luciferase reporter assay.

**Results:**

CircMAPK9 and PPM1A were upregulated and miR-140-3p was downregulated in RA patients and FLSs from RA patients (RA-FLSs). CircMAPK9 silence suppressed cell proliferation, migration, invasion, inflammatory response, and promoted apoptosis in RA-FLSs. MiR-140-3p was a target of circMAPK9, and miR-140-3p downregulation attenuated the effects of circMAPK9 knockdown on cell progression and inflammatory response in RA-FLSs. PPM1A was targeted by miR-140-3p, and circMAPK9 could regulate PPM1A expression by sponging miR-140-3p. Furthermore, miR-140-3p could impede cell biological behaviors in RA-FLSs via targeting PPM1A.

**Conclusion:**

CircMAPK9 knockdown might inhibit cell proliferation, migration, invasion, inflammatory response, and facilitate apoptosis in RA-FLSs via regulating miR-140-3p/PPM1A axis, offering a new mechanism for the comprehension of RA development and a new insight into the potential application of circMAPK9 in RA treatment.

## Introduction

Rheumatoid arthritis (RA) is a common chronic autoimmune disorder that mainly influences the synovial joints [1, 2]. Fibroblast-like synoviocytes (FLSs), one class of dominating cells in synovial tissues, are reported to serve a vital role in the pathogenesis of RA [3, 4]. Although several treatment options are available for the management of RA patients, there is no cure for RA [5]. Thus, it is essential to explore the mechanism of FLSs progression in order to find new targets for RA treatment.

Circular RNAs (circRNAs) are a special class of noncoding RNAs possessing continuous covalently closed loops that are produced via back-splicing of precursor mRNAs [6, 7]. Increasing evidence supported that circRNAs are concerned with the onset and development of multiple human diseases [8]. Meanwhile, numerous circRNAs played vital part in the progression of autoimmune diseases, including RA [9]. CircRNA mitogen-activated protein kinase (circMAPK9), also known as hsa_circ_0001566, is derived from back-splicing of MAPK9 transcript and has been reported to be highly expressed in peripheral blood mononuclear cells (PBMCs) from RA patients [10], whereas the exact role and regulatory mechanism of circMAPK9 in FLSs progression is indistinct.

MicroRNAs (miRNAs) are defined as small noncoding molecules that can regulate gene expression through combining with the 3′untranslated regions (3′UTRs) of target mRNAs [11]. Generally, circRNAs are known to work as miRNA molecular sponges to inhibit miRNA activity by competitively binding to miRNAs [12]. Plentiful miRNAs have been found to be dysregulated and may serve as biomarkers or therapeutic targets in RA [13–15]. Furthermore, miR-140-3p abundance was declined in synovial tissue and FLSs from RA patients (RA-FLSs) and from mice in arthritis models, and miR-140-3p overexpression in FLSs inhibited cell proliferation and migration [16]. Moreover, Lee et al. reported that protein phosphatase magnesium-dependent 1A (PPM1A) was involved in the development of RA [17]. Nevertheless, the relationships among circMAPK9, miR-140-3p, and PPM1A in the pathogenesis of RA are undiscovered.

In this research, circMAPK9 abundance was measured in RA patients and RA-FLSs. Then, we explored the effects of circMAPK9 on cell growth, transferability, and inflammation in RA-FLSs. Besides, we uncovered the regulatory network of circMAPK9/miR-140-3p/PPM1A in RA-FLSs.

## Materials and methods

### Patient tissue collection

RA synovial tissues were collected from RA patients (*n* = 22) who underwent knee replacement surgery. Normal synovial tissues were obtained from patients with traumatic knee and no history of autoimmune diseases (*n* = 22). All subjects were recruited from Ganzhou People’s Hospital, The Affiliated Ganzhou Hospital of Nanchang University, and they all signed the written informed consent. After surgical resection, these tissues were preserved at – 80 °C until usage. This research was permitted by the Ethical Committee of Ganzhou People’s Hospital, The Affiliated Ganzhou Hospital of Nanchang University. The clinical characteristics of RA and trauma patients are listed in Table 1.

**Table 1:** Clinical characteristics of rheumatoid arthritis (RA) and trauma patients

Clinical data	RA patients	Trauma patients
Number of subjects	22	22
Sex (male/female)	10/12	8/14
Age (years)	51.35 *±* 15.25	46.54 *±* 11.32
Disease duration (years)	8.65 ± 5.68	/
Tender joints^a^	10.35 ± 9.56	/
Swollen joints^a^	11.68 ± 9.86	/
CRP (mg/dl)	34.25 ± 23.65	/
ESR (mm/h)	42.35 ± 25.68	/
ACPA (RU/ml)	712.65 ± 512.34	/
DAS28	5.48 ± 1.95	/
DMARD (n)	14	/
NSAID (n)	9	/
Corticosteroid (n)	12	

*CRP* C-reactive protein, *ESR* erythrocyte sedimentation rate, *ACPA* anti-citrullinated protein antibodies, *DAS28* disease activity score, *DMARD* disease-modifying anti-rheumatic drug, *NSAID* non-steroidal anti-inflammatory drug, *n* number of patients

^a^Twenty-eight joints were assessed for tenderness, and twenty-eight were assessed for swelling

### Cell culture

Fibroblast-like synoviocytes from RA patients (RA-FLSs) or healthy subjects (H-FLSs) were separated as formerly mentioned [18]. Briefly, synovial tissue samples were cut into small debris and digested using 2 mg/mL of collagenase (type II, Thermo Fisher Scientific, Waltham, MA, USA) at 37 °C for 2 h to isolate synoviocytes. FLSs were cultured in HFLS growth medium (Cell Applications, San Diego, CA, USA) plus 10% fetal bovine serum (FBS, HyClone, Logan, UT, USA) in a 37 °C incubator containing 5% CO_2_. FLSs were separated from all healthy donors and RA patients for detecting the circMAPK9 expression. Two sets of RA-FLSs were selected for functional assays. Set 1 of RA-FLSs was acquired from three random-selected RA patients and set 2 of RA-FLS was acquired from another three random-selected RA patients. Then, the same number of RA-FLSs was mixed from these three RA patients. The RA-FLSs were cultured, and set 1 of RA-FLSs was utilized for mechanistic investigation. In these experiments, cells at passage 3 were used.

### Quantitative real-time polymerase chain reaction (qRT-PCR)

Total RNA was extracted with TRIzol (Invitrogen, Carlsbad, CA, USA). Next, cDNA was synthesized from RNA reverse transcription using M-MLV Reverse Transcriptase kit (Thermo Fisher Scientific) or TaqMan microRNA Reverse Transcription Kit (Applied Biosystems, Foster City, CA, USA). Then, qRT-PCR were implemented on ABI Prism 7900HT Detection System (Applied Biosystems) employing SYBR Master Mix (Takara, Tokyo, Japan) and specific primers (Sangon, Shanghai, China) with the amplification protocol: 95 °C for 5 min, 40 cycles of 95 °C for 30 s, 55 °C for 45 s, and 72 °C for 30 s. The relative RNA expression was calculated with 2^−ΔΔCt^ method. The primers were exhibited as follows: circMAPK9 (F, 5′-CATGGAGCTGGATCATGAAA-3′; R, 5′-AGGTTGAGTCTGCCACTTGC-3′), MAPK9 (F, 5′-TACGTGGTGACACGGTACTACC-3′; R, 5′-CACAACCTTTCACCAGCTCTCC-3′), miR-140-3p (F, 5′-CAGTGCTGTACCACAGGGTAGA-3′; R, 5′-TATCCTTGTTCACGACTCCTTCAC-3′), PPM1A (F, 5′-GAAGAAGGAGGCAGAGTTGGAC-3′; R, 5′-GGATGTTCTCACTCGCTAATGTG-3′), U6 (F, 5′-CTCGCTTCGGCAGCACA-3′; R, 5′-AACGCTTCACGAATTTGCGT-3′), and β-actin (F, 5′-GTCACCGGAGTCCATCACGAT-3′; R, 5′-TCACCAACTGGGACGACATG-3′). β-actin and U6 were served as the inner references.

### RNase R treatment

For detecting the stability of circMAPK9, total RNA (2 μg) was reacted with RNase R (3 U/μg, Geneseed, Guangzhou, China) at 37 °C for 0.5 h to digest linear RNA. After that, the treated RNA was used for qRT-PCR to survey the RNA abundance of circMAPK9 and linear MAPK9.

### Cell transfection

The small interfering RNA (siRNA) against circMAPK9 (si-circMAPK9#1, si-circMAPK9#2 or si-circMAPK9#3), miR-140-3p mimic (miR-140-3p) or miR-140-3p inhibitor (anti-miR-140-3p), and corresponding controls (si-NC, miR-NC or anti-miR-NC) were acquired from Ribobio (Guangzhou, China). PPM1A overexpression vector (PPM1A) based on the pcDNA3.1 vector and the empty pcDNA3.1 vector (vector) were also acquired from Ribobio. Cell transfection was performed through introducing the above oligonucleotides (50 nM) or vectors (40 ng) into RA-FLSs (1 × 10^5^) at 70–80% confluence via the Lipofectamine 3000 reagent (Invitrogen; 1 μL/each well).

### 3-(4,5-Dimethylthiazol-2-yl)-2,5-diphenyltetrazolium bromide (MTT) assay

For proliferation detection, 1 × 10^4^ RA-FLSs were placed into 96-well plates and incubated for 72 h. Then, the cells were shifted into fresh medium plus MTT (0.5 mg/mL, Beyotime, Shanghai, China). After incubation for another 4 h, the medium was changed to 100 μL dimethyl sulfoxide (DMSO; Solarbio, Beijing, China). The absorbance at 570-nm wavelength was examined via a microplate reader (Molecular Devices, Sunnyvale, CA, USA).

### Flow cytometry

Flow cytometry analysis was executed for the detection of cell apoptosis and cycle distribution. For cell apoptosis detection, Annexin V-fluorescein isothiocyanate (FITC) Apoptosis Detection Kit (BD Biosciences, San Jose, CA, USA) was utilized. Briefly, following transfection for 48 h, 1 × 10^5^ RA-FLSs were collected and re-suspended in Annexin binding buffer. Then, cells were stained with 5 μL Annexin V-FITC and 5 μL propidium iodide (PI) in a dark place at 4 °C for 15 min. Subsequently, the apoptotic cells were analyzed by a flow cytometer (BD Biosciences). For cell cycle distribution detection, RA-FLSs (1 × 10^5^) after 48-h transfection were collected and re-suspended in PBS, then added with PI (BD Biosciences) staining solution in dark place at 37 °C for 10 min after 70% ethanol fixation. The distribution of different cell cycle phases (G0/G1, S and G2/M) was assessed utilizing a flow cytometer (BD Biosciences).

### Transwell assay

Transwell chambers (Costar, Corning, NY, USA) were used to detect cell migratory and invasive abilities. 5 × 10^4^ RA-FLSs or 1 × 10^4^ RA-FLSs after 48-h transfection was re-suspend in 200 μL of serum free HFLS medium and then added into the upper chamber coated with or without Matrigel (BD Biosciences) to identify cell invasion and migration, respectively. The lower chamber was added with 0.6 mL complete HFLS medium with 10% FBS. The non-migrating or non-invading cells were removed after incubation for 24 h, and migratory and invasive cells on the bottom membrane were fastened by paraformaldehyde (4%, Beyotime) and dyed by crystal violet solution (0.1%, Beyotime) for 30 min. The migratory or invasive cells were observed and counted using a microscope (Olympus, Tokyo, Japan) at × 100 magnification.

### Western blot

RIPA lysis buffer (Beyotime) and BCA protein assay kit (Beyotime) were utilized for total protein extraction and quantification, respectively. Then, equal amount of protein (20 μg/lane) was segregated by SDS-PAGE gel and then shifted into PVDF membrane (Solarbio). After blocked in 5% non-fat milk for 1 h, the membrane was probed with specific primary antibodies at 4 °C overnight, and subsequently probed with HRP-conjugated secondary antibody (1:20,000, ab205718 or ab205719, Abcam, Cambridge, MA, USA) for 2 h. The blots were exposed to enhanced BeyoECL Moon (Beyotime), and the bands density was assessed via Image J software (NIH, Bethesda, MD, USA). The primary antibodies obtained from Abcam included anti-B-cell lymphoma-2 antibody (Bcl-2; 1:1000, ab32124), anti-BCL2-associated X protein antibody (anti-Bax; 1:1000, ab32503), anti-matrix metalloproteinase 2 (MMP2) (1:1000, ab86607) antibody, anti-MMP9 (1:1000, ab137867) antibody, anti-PPM1A (1:500, ab14824) antibody, and β-actin antibody (1:5000; ab6276). Relative protein expression was normalized by internal reference β-actin.

### Enzyme-linked immunosorbent assay (ELISA)

ELISA was performed to inspect the secretion of tumor necrosis factor alpha (TNF-α), interleukin (IL)-1β and IL-6 in culture supernatant of RA-FLSs. Briefly, 1 × 10^5^ RA-FLSs were plated into 12-well plates. Seventy-two hours post-transfection, culture supernatant of each group was collected. The concentrations of TNF-α, IL-1β, and IL-6 in culture supernatant were examined using corresponding Human TNF-α, IL-1β, or IL-6 ELISA Kit (Abcam).

### Dual-luciferase reporter analysis

The sequence of circMAPK9 or PPM1A 3′UTR carrying wild-type (WT) or mutant (MUT) complementary sites of miR-140-3p was cloned into pmirGLO vector (Promega, Madison, WI, USA) to generate circMAPK9 WT and circMAPK9 MUT, PPM1A 3′UTR WT, and PPM1A 3′UTR MUT. The sites of circMAPK9 and PPM1A were mutated using the QuickChange XL site-directed mutagenesis kit (Stratagene, La Jolla, CA, USA). RA-FLSs (1 × 10^5^ cells) were transfected with the constructed luciferase vector (20 ng) and miR-NC or miR-127-5p (20 nM) for 48 h. The luciferase activity was examined by Dual-Lumi™ Luciferase Assay Kit (Beyotime), followed by normalization to the Renilla luciferase.

### Statistical analysis

Statistical analysis was executed using GraphPad Prism 6 (GraphPad Inc., La Jolla, CA, USA). All data from at least 3 independent biological replications were displayed as mean ± standard deviation (SD). Difference was analyzed using Student’s *t*-test (between 2 groups) or one-way analysis of variance followed by Tukey test (among multiple groups) in specific circumstances. Statistical significance was considered when *P*-value < 0.05.

## Results

### CircMAPK9 was upregulated in RA patients and RA-FLSs

**Fig. 1:**
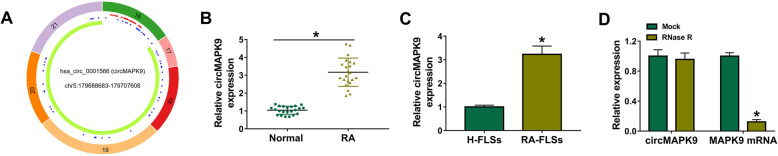
CircMAPK9 expression was increased in RA patients and RA-FLSs. **A** Schematic illustration exhibiting the circularization of MAPK9 exons 16 to 21 forming circMAPK9. **B** The expression of circMAPK9 was determined by qRT-PCR analysis in synovial tissues from RA patients (*n* = 22) and normal patients (*n* = 22). **C** The abundance of circMAPK9 in RA-FLSs and control H-FLSs was measured via qRT-PCR. **D** The expression of circMAPK9 and linear mRNA (MAPK9) was examined via qRT-PCR after RNase R treatment. *P < 0.05

CircMAPK9 (hsa_circ_0001566) was located on chr5:179688683-179707608 of chromosome and derived from exon 16-21 of MAPK9 genome (Fig. 1A). To explore the potential roles of circMAPK9 in RA, its expression pattern was detected by qRT-PCR in synovial tissues from RA patients (*n* = 22) and normal patients (*n* = 22). The results showed that circMAPK9 abundance was greatly increased in RA patients compared to normal patients (Fig. 1B). Then, the expression of circMAPK9 in RA-FLSs or normal subjects (H-FLSs) was measured. The results demonstrated that circMAPK9 level was higher in RA-FLSs more than triple than control group (Fig. 1C). Furthermore, the stability of circRNA was evaluated by RNase R digestion assay. As displayed in Fig. 1D, linear mRNA (MAPK9) was obviously decreased after digestion by RNase R while circMAPK9 expression was not affected, indicating the cyclic structure of circMAPK9. These data indicated that increased expression of circMAPK9 might be associated with RA progression.

### Knockdown of circMAPK9 inhibited cell proliferation, migration, invasion, inflammation, and promoted apoptosis in RA-FLSs

**Fig. 2:**
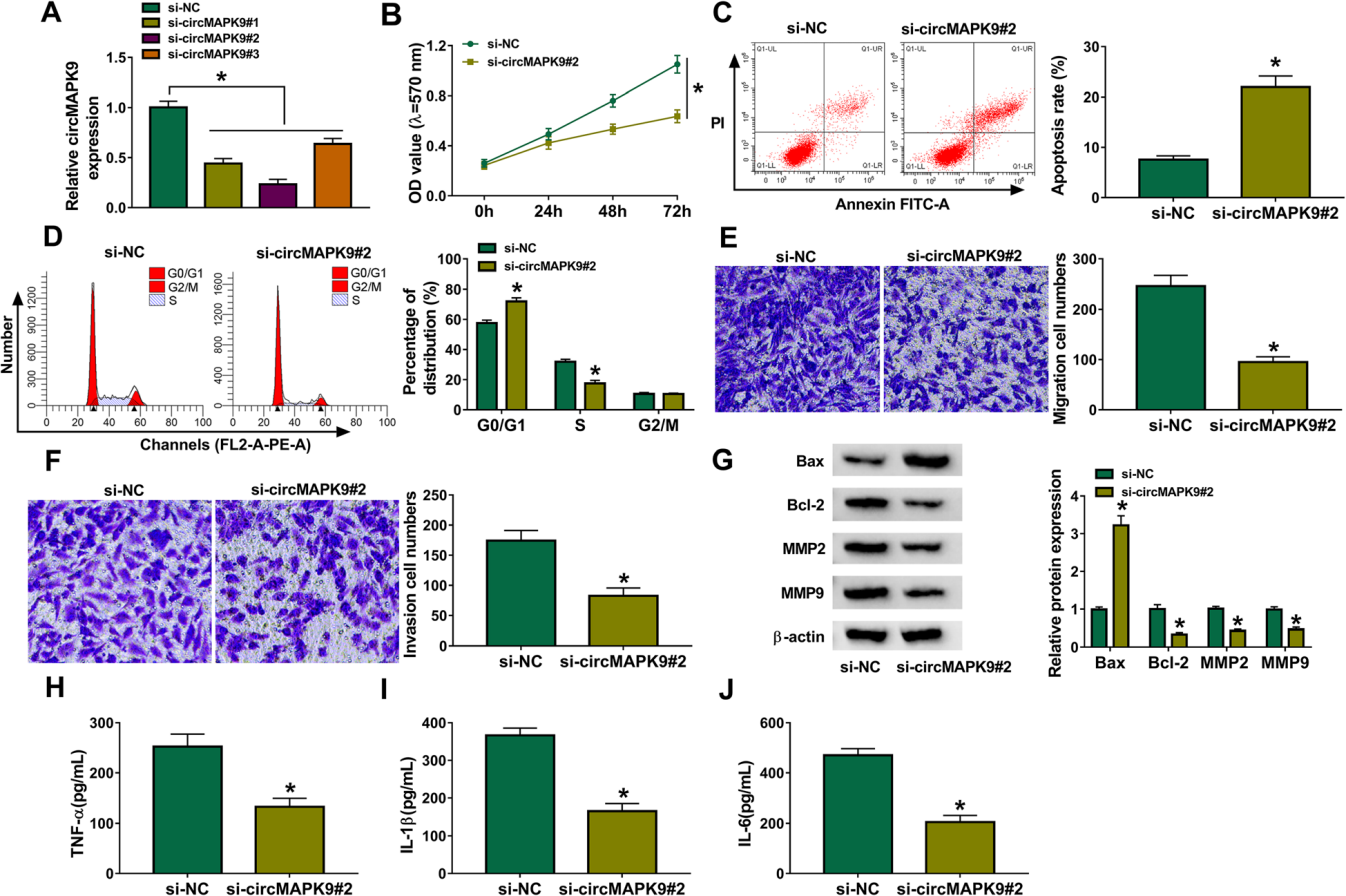
The influence of circMAPK9 on cell proliferation, apoptosis, cycle distribution, migration, invasion, and inflammatory response in RA-FLSs. **A** Knockdown efficiency of circMAPK9 was detected by qRT-PCR in RA-FLSs with transfection of si-NC, si-circMAPK9#1, si-circMAPK9#2, or si-circMAPK9#3. **B**–**J** RA-FLSs were transfected with si-NC or si-circMAPK9#2. **B** Cell proliferation was assessed by MTT assay. **C**, **D** Cell apoptosis and cycle distribution were measured via flow cytometry. **E**, **F** Cell migration and invasion were examined via transwell analysis. **G** Western blot assay was conducted to measure the protein levels of Bax, Bcl-2, MMP2, and MMP9. **H**–**J** The inflammatory cytokine secretion (TNF-α, IL-1β, IL-6) was detected via ELISA after transfection. *P < 0.05

To study the effect of circMAPK9 on RA progression, loss-of-function experiments were performed in RA-FLSs transfected with siRNAs to knock down circMAPK9. As displayed in Fig. 2A, relative to si-NC group, the expression of circMAPK9 was signally declined in RA-FLSs transfected with si-circMAPK9#1, si-circMAPK9#2, or si-circMAPK9#3, especially in si-circMAPK9#2 group. Therefore, si-circMAPK9#2 was chosen for further study. Next, the impacts of circMAPK9 on cell proliferation, apoptosis, cycle distribution, and invasiveness were investigated. MTT assay indicated that knockdown of circMAPK9 restrained cell proliferation in RA-FLSs (Fig. 2B). Flow cytometry assay showed that circMAPK9 silence evidently promoted cell apoptosis and induced cell cycle arrest at G0/G1 phase in RA-FLSs (Fig. 2C, D). Moreover, circMAPK9 deficiency markedly restrained the migratory and invasive abilities of RA-FLSs using transwell analysis (Fig. 2E, F). Besides, western blot assay exhibited that circMAPK9 interference significantly increased the level of pro-apoptotic protein Bax, and decreased the expression of anti-apoptotic protein Bcl-2, migration and invasion-related proteins (MMP2 and MMP9), further supporting the effects of circMAPK9 silence on cell apoptosis and invasiveness (Fig. 2G). Additionally, the inflammatory response was analyzed in RA-FLSs via ELISA, which presented that circMAPK9 knockdown visibly reduced the secretion of pro-inflammatory cytokines (TNF-α, IL-1β, and IL-6) in RA-FLSs (Fig. 2H–J). These results indicated that circMAPK9 downregulation could suppress cell proliferation, migration, invasion, inflammatory response, and accelerate apoptosis of RA-FLSs.

### CircMAPK9 acted as a sponge of miR-140-3p

**Fig. 3:**
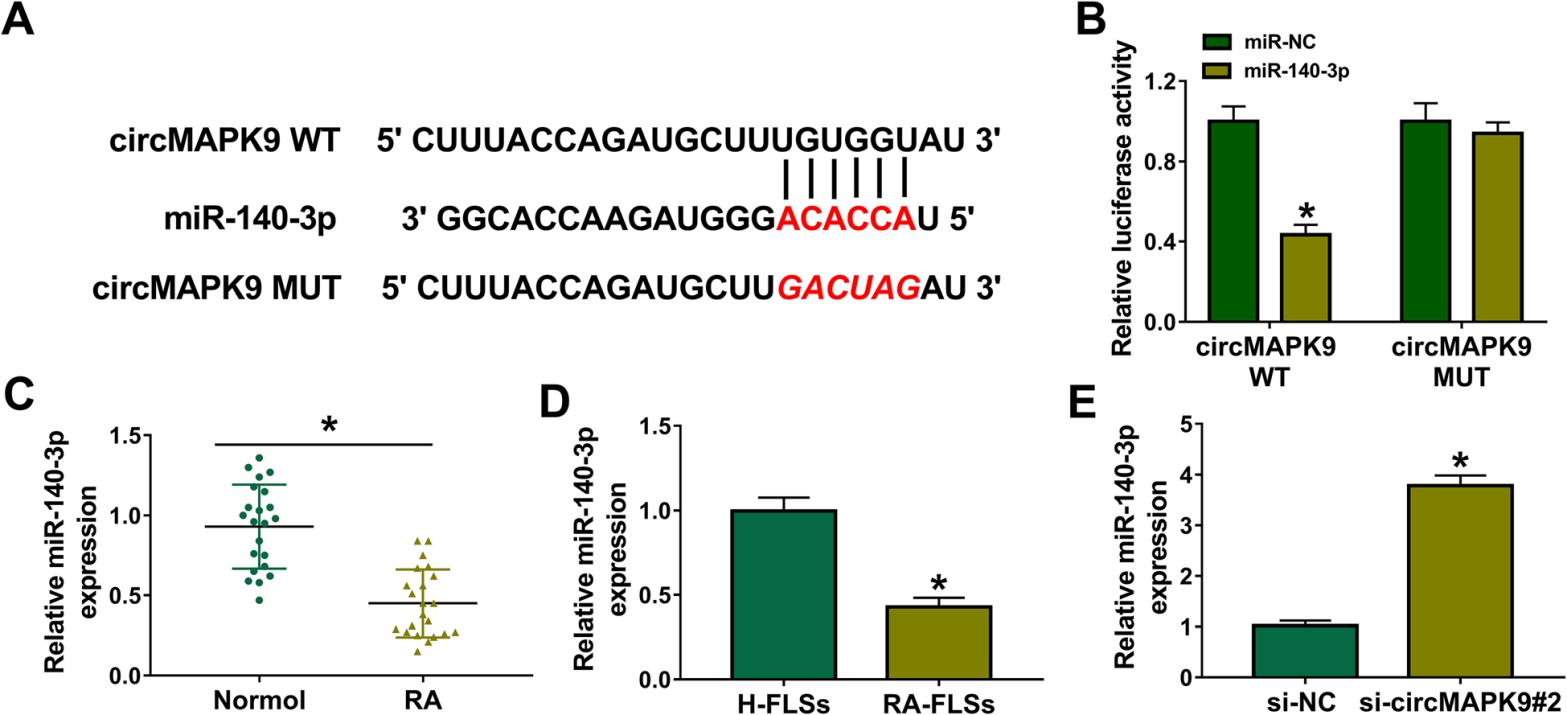
CircMAPK9 directly interacted with miR-140-3p. **A** The putative binding sites between circMAPK9 and miR-140-3p were predicted by CircInteractome. **B** Dual-luciferase reporter assay was performed in RA-FLSs with co-transfection of circMAPK9 WT or circMAPK9 MUT and miR-NC or miR-140-3p. **C** The expression of miR-140-3p was examined by qRT-PCR in synovial tissues from RA patients (*n* = 22) and normal patients (*n* = 22). **D** The level of miR-140-3p was measured in H-FLSs and RA-FLSs by qRT-PCR. **E** The enrichment of miR-140-3p was analyzed via qRT-PCR in RA-FLSs with transfection of si-NC or si-circMAPK9#2. *P < 0.05

To analyze the potential mechanism of circMAPK9, the potential target miRNAs of circMAPK9 were predicted using CircInteractome (https://circinteractome.nia.nih.gov/mirna_target_sites.html). The results showed that miR-140-3p had putative binding sequence for circMAPK9 (Fig. 3A). To validate the relationship between circMAPK9 and miR-140-3p, dual-luciferase reporter assay was performed through constructing circMAPK9 WT and circMAPK9 MUT. The results displayed that miR-140-3p overexpression remarkably decreased the luciferase activity of circMAPK9 WT but not that of circMAPK9 MUT when the binding sites were mutated (Fig. 3B). Furthermore, the level of miR-140-3p in RA patients and RA-FLSs was observed. Results showed that the abundance of miR-140-3p was evidently decreased in synovial tissues from RA patients and RA-FLSs (Fig. 3C, D). In addition, the expression of miR-140-3p was promoted after knockdown of circMAPK9 (Fig. 3E). The above evidence verified that miR-140-3p was a direct target of circMAPK9.

### MiR-140-3p knockdown reversed the effects of si-circMAPK9#2 on cell proliferation, migration, invasion, and inflammatory response in RA-FLSs

**Fig. 4:**
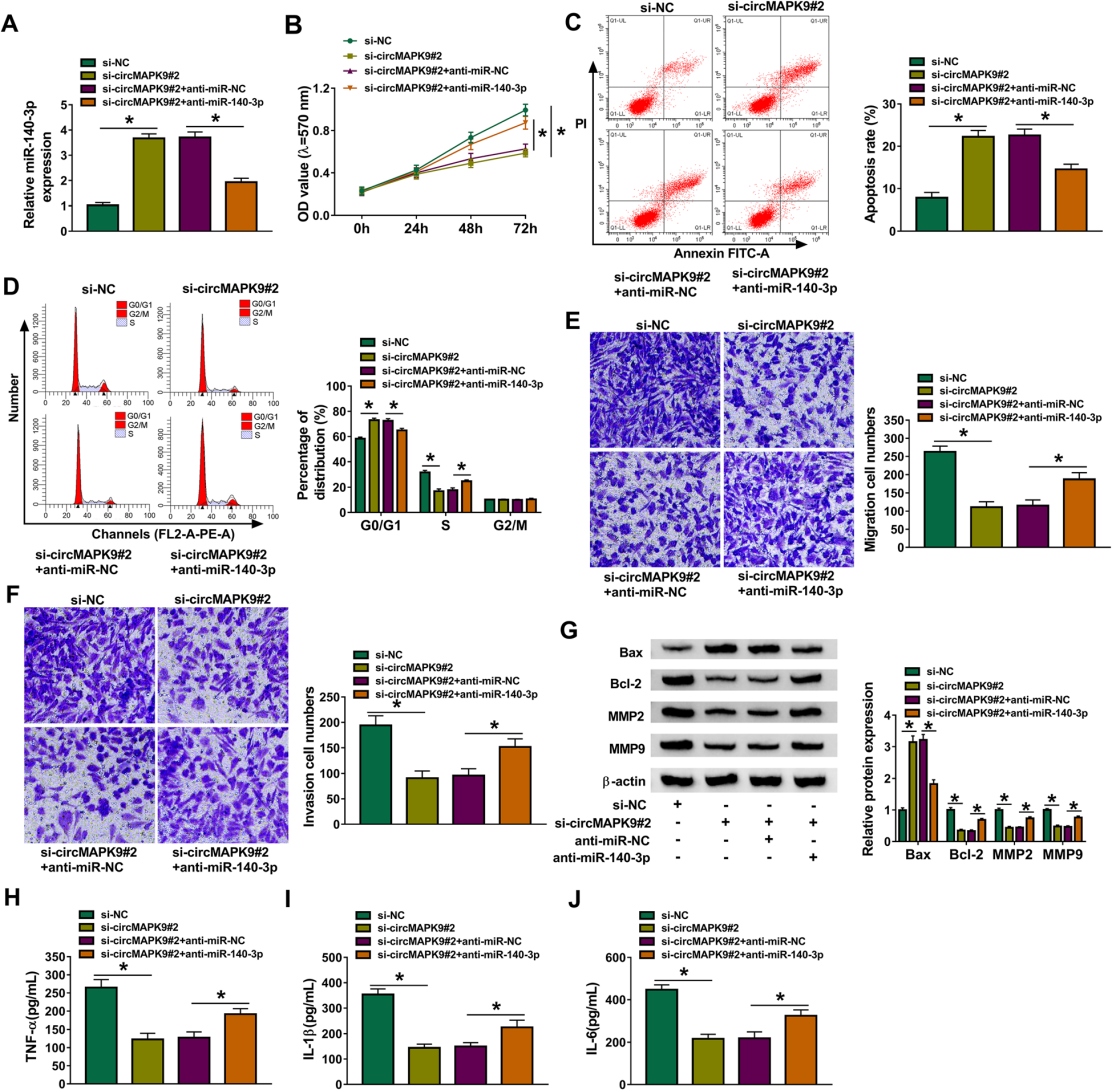
CircMAPK9 exerted its functions by sponging miR-140-3p in RA-FLSs. RA-FLSs were transfected with si-NC, si-circMAPK9#2, si-circMAPK9#2 + anti-miR-NC, or si-circMAPK9#2 + anti-miR-140-3p. **A** The level of miR-140-3p was examined by qRT-PCR. **B** MTT assay was conducted to evaluate cell proliferation. **C**, **D** Flow cytometry analysis was employed to measure the apoptosis rate and cell cycle distribution. **E**, **F** Transwell assay examined cell migration and invasion. **G** The protein levels of Bax, Bcl-2, MMP2, and MMP9 were determined by western blot. **H**–**J** The secretion of TNF-α, IL-1β, and IL-6 was tested by ELISA. *P < 0.05

To explore whether the biological function of circMAPK9 in RA was mediated by miR-140-3p, rescue experiments were performed. RA-FLSs were transfected with si-NC, si-circMAPK9#2, si-circMAPK9#2 + anti-miR-NC, or si-circMAPK9#2 + anti-miR-140-3p. The abundance of miR-140-3p was increased via circMAPK9 knockdown, which was reversed by downregulating miR-140-3p (Fig. 4A). Furthermore, the inhibitory effect of circMAPK9 downregulation on cell proliferation and the promoting effects of circMAPK9 silence on apoptosis and cell cycle arrest at G0/G1 phase were all mitigated by downregulation of miR-140-3p (Fig. 4B–D). Moreover, miR-140-3p inhibition abated the effect of circMAPK9 silence-mediated suppression on cell migration and invasion (Fig. 4E, F). Correspondingly, the enhancement of Bax expression and decrease of Bcl-2, MMP2, and MMP9 were all relieved by miR-140-3p deficiency in circMAPK9-silenced RA-FLSs (Fig. 4G). Additionally, the reduction of TNF-α, IL-1β, and IL-6 levels in RA-FLSs caused by si-circMAPK9#2 transfection was restored by co-transfection with anti-miR-140-3p (Fig. 4H–J). Taken together, these data illustrated that circMAPK9 exerted its biological function in RA-FLSs by sponging miR-140-3p.

### PPM1A was identified to be a target of miR-140-3p

**Fig. 5:**
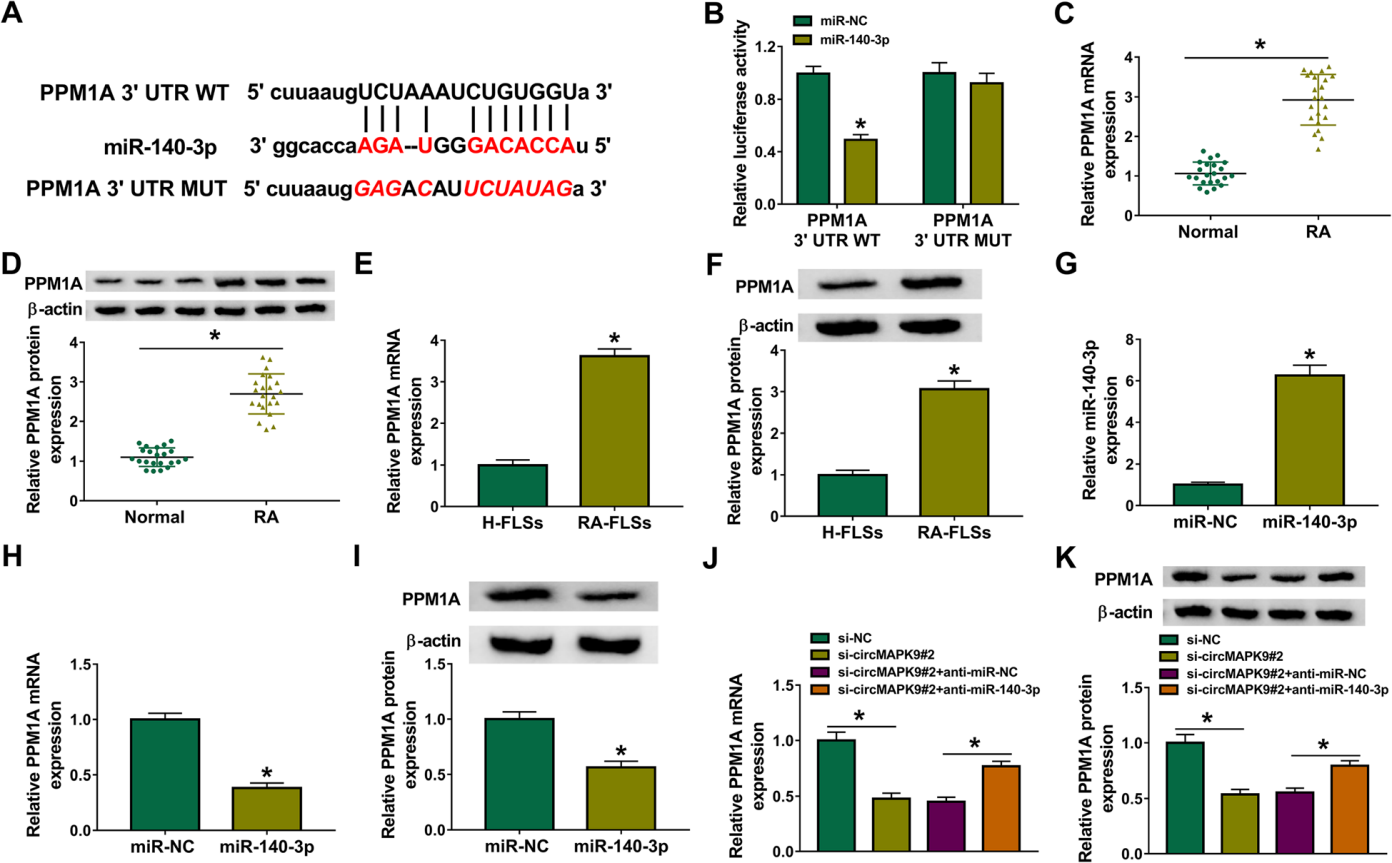
PPM1A was a downstream target of miR-140-3p. **A** The predicted binding sequence of miR-140-3p and PPM1A was shown. **B** Relative luciferase activity was determined by dual-luciferase reporter assay in RA-FLSs co-transfected with PPM1A 3′UTR WT and PPM1A 3′UTR MUT and miR-NC or miR-140-3p. **C**, **D** The mRNA and protein expression of PPM1A was examined in synovial tissues from RA patients (*n* = 22) and normal patients (*n* = 22) by qRT-PCR and western blot assays, respectively. **E**, **F** PPM1A mRNA and protein levels were determined in RA-FLSs and control H-FLSs by qRT-PCR and western blot assays, respectively. **G** The enrichment of miR-140-3p was examined via qRT-PCR in RA-FLSs with transfection of miR-NC or miR-140-3p. **H**, **I** The mRNA and protein levels of PPM1A in RA-FLSs with transfection of miR-NC or miR-140-3p were tested by qRT-PCR and western blot assays, respectively. **J**, **K** PPM1A mRNA and protein levels were detected in RA-FLSs with transfection of si-NC, si-circMAPK9#2, si-circMAPK9#2 + anti-miR-NC, or si-circMAPK9#2 + anti-miR-140-3p by qRT-PCR and western blot assays, respectively. *P < 0.05

To further analyze the regulatory network, starBase v2.0 online website (http://starbase.sysu.edu.cn/agoClipRNA.php?source=mRNA) was utilized to search for the potential target mRNAs of miR-140-3p. The prediction result suggested that PPM1A 3′UTR shared binding sites for miR-140-3p (Fig. 5A), suggesting that PPM1A could possibly interact with miR-140-3p. To confirm this assumption, PPM1A 3′UTR WT and PPM1A 3′UTR MUT were constructed, and then dual-luciferase reporter assay was implemented. The results indicated that miR-140-3p introduction significantly decreased the luciferase activity of PPM1A 3′UTR WT, whereas little change was observed in the luciferase activity of PPM1A 3′UTR MUT (Fig. 5B). The qRT-PCR and western blot assays results displayed that PPM1A mRNA and protein abundance was markedly upregulated in synovial tissues from RA patients and RA-FLSs (Fig. 5C–F). Next, the relationships among circMAPK9, miR-140-3p, and PPM1A were explored. The result of qRT-PCR showed that the abundance of miR-140-3p was strikingly increased in RA-FLSs transfected with miR-140-3p (Fig. 5G), indicating the high transfection efficacy of miR-140-3p. Meanwhile, overexpression of miR-140-3p visibly inhibited the mRNA and protein expression of PPM1A (Fig. 5H, I). Moreover, circMAPK9 silence markedly reduced the mRNA and protein levels of PPM1A, which could be reversed by downregulating miR-140-3p (Fig. 5J, K), indicating that circMAPK9 upregulated PPM1A expression by downregulating miR-140-3p. These data collectively demonstrated that PPM1A was a downstream target of miR-140-3p, and circMAPK9 could positively regulate PPM1A expression by sponging miR-140-3p.

### MiR-140-3p overexpression could suppress cell progression and inflammatory response via downregulating PPM1A in RA-FLSs

**Fig. 6:**
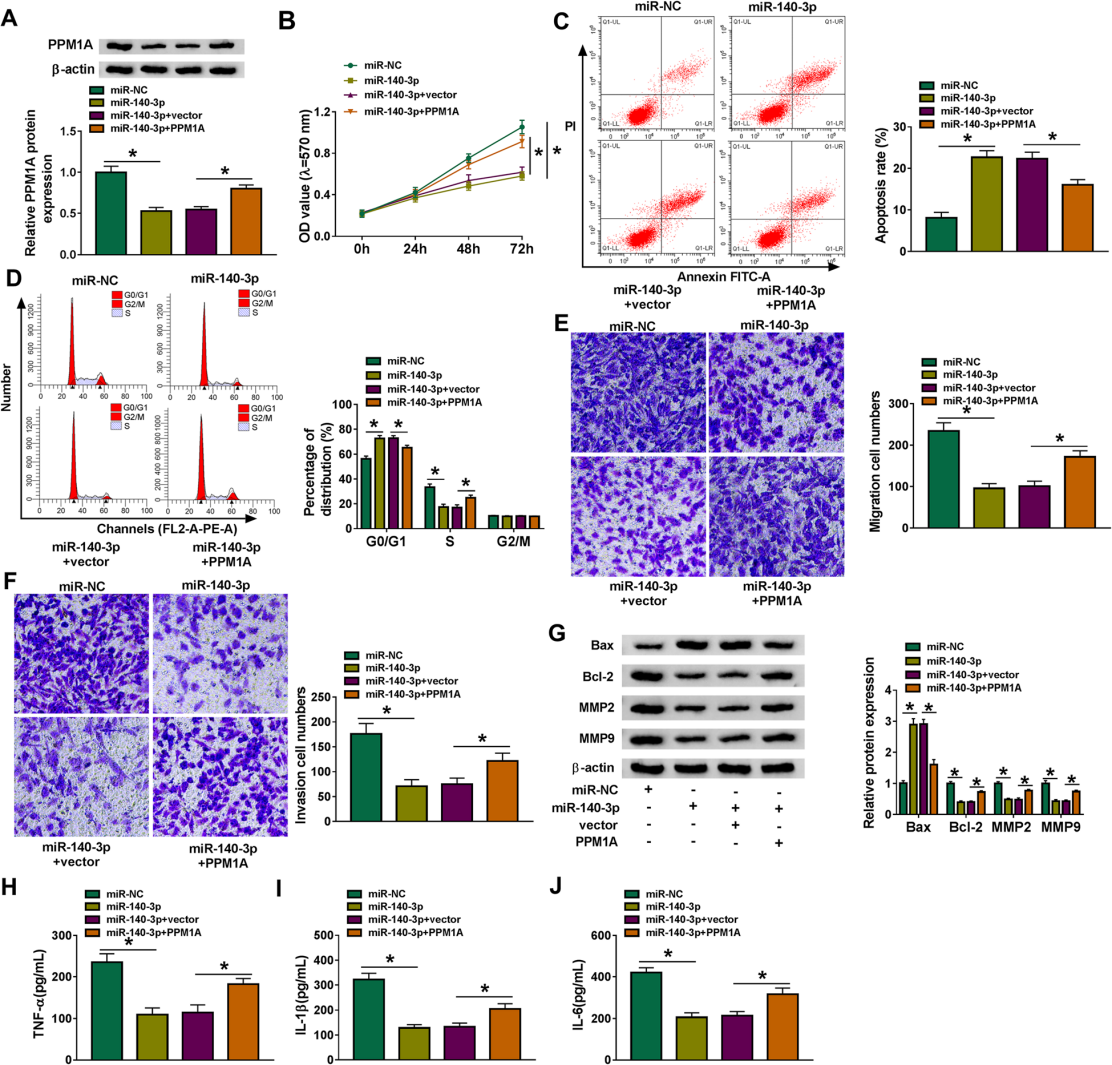
The influence of miR-140-3p and PPM1A on cell malignant progression and inflammatory response in RA-FLSs. RA-FLSs were transfected with miR-NC, miR-140-3p, miR-140-3p + vector, or miR-140-3p + PPM1A. **A** Western blot assay was performed to detect the protein expression of PPM1A. Cell proliferation (**B**), apoptosis (**C**) and cycle distribution (**D**), migration (**E**), and invasion (**F**) were measured by MTT assay, flow cytometry analysis, or transwell assay, respectively. **G** Western blot was carried out to examine the protein expression of Bax, Bcl-2, MMP2, and MMP9. **H**–**J** The levels of TNF-α, IL-1β, and IL-6 were analyzed by ELISA. *P < 0.05

To explore whether miR-140-3p exerted its biological functions in RA-FLSs by targeting PPM1A, RA-FLSs were transfected with miR-NC, miR-140-3p, miR-140-3p + vector, or miR-140-3p + PPM1A. Western blot assay showed that miR-140-3p overexpression overtly inhibited the protein expression of PPM1A, which was restored via addition of PPM1A overexpression vector (Fig. 6A). Moreover, overexpression of miR-140-3p repressed cell proliferation and inducing cycle arrest at G0/G1 phase and apoptosis, which could be reversed by introduction of PPM1A (Fig. 6B–D). Also, PPM1A upregulation evidently overturned the repressive effect of miR-140-3p overexpression on cell migration and invasion (Fig. 6E, F). Furthermore, miR-140-3p addition increased the protein level of Bax and decreased the protein expression of Bcl-2 and MMP2 as well as MMP9, whereas these effects were abated by upregulating PPM1A (Fig. 6G). Additionally, the protein levels of TNF-α, IL-1β, and IL-6 were significantly reduced in RA-FLSs after transfection with miR-140-3p, while co-transfection with PPM1A overexpression vector mitigated these effects (Fig. 6H–J). Altogether, these data proved that miR-140-3p could inhibit cell proliferation, migration, invasion, inflammatory response, and facilitate apoptosis of RA-FLSs by targeting PPM1A.

## Discussion

RA is a common form of inflammatory multisystem disease with undiscovered etiology [2]. CircMAPK9 was highly expressed in PBMCs from RA patients and might act as a possible diagnostic biomarker for RA [10]. FLSs play a pivotal role in RA etiology by regulating inflammatory response and cartilage destruction [19, 20]. Thereby, investigating the tumor-like biologic behaviors of RA-FLSs is indispensable to develop novel therapies for RA patients. Meanwhile, noncoding RNAs (ncRNAs) including circRNAs might serve as promising biomarkers for RA [21]. In this report, we aimed to study the biological role of circMAPK9 and explore the underlying mechanism in the advancement of RA-FLSs. Through the verification of functional experiment, we first clarified that circMAPK9 knockdown repressed proliferation, invasiveness, and inflammation of RA-FLSs via circMAPK9/miR-140-3p/PPM1A regulatory network.

The deregulation of circRNAs is identified to be closely related to the occurrence and development of autoimmune diseases including RA [22]. For instance, circ_0088036 promoted the proliferative and migratory capacities of FLSs via the circ0088036/miR-140-3p/SIRT1 axis in RA [23]. In keeping with previously report [10], we also verified that circMAPK9 level was enhanced in synovial tissues from RA patients and RA-FLSs. Thus, we speculated that the disordered level of circMAPK9 might be connected with RA evolution. Through implementing loss-of-function experiment in RA-FLSs, it was evidenced that circMAPK9 silence could repress cell proliferation, migration, invasion, and accelerated apoptosis of RA-FLSs. Many cytokines are associated with RA progression, including TNF-α, IL-1, IL-6, and IL-17 [24, 25]. Furthermore, this report also attested that circMAPK9 knockdown lessened the inflammatory response of RA-FLSs by decreasing the release of TNF-α, IL-1β, and IL-6. Hence, we deemed that circMAPK9 might contribute to RA malignant development by facilitating cell proliferation, migration, invasion, inflammatory response, and hindering cell apoptosis of RA-FLSs.

Accumulating reports have revealed that circRNAs could modulate the progression of multifarious diseases via acting as miRNA sponges [26]. As well, miRNAs have been certified to serve pivotal part in FLSs of RA [27]. For instance, miR-20a was involved in the modulation of pro-inflammatory cytokines release by controlling ASK1 expression in RA-FLSs [28]. To validate whether miRNAs were implicated in circMAPK9-mediated RA evolution, CircInteractome database was applied to forecast the possible miRNAs of circMAPK9. The prediction result indicated that miR-140-3p was targeted by circMAPK9 in RA-FLSs, and the dual-luciferase reporter assay verified the interacting effect between them furtherly. Previous studies have demonstrated the suppressive role of miR-140-3p in the progress of bladder cancer [29], colorectal cancer [30], and so on. Yin et al. pointed out that the decline of miR-140-3p was correlated with increased osteoarthritis severity [31]. Moreover, Zhong et al. illuminated the participation of miR-140-3p in the proliferative and migratory processes of RA-FLSs via SIRT1 in RA [23]. In this research, low expression of miR-140-3p was observed in synovial tissues from RA patients and RA-FLSs, which was in agreement with previous work [16]. Simultaneously, miR-140-3p could restrain cell propagation, transferability, and inflammatory response of RA-FLSs. Besides, miR-140-3p silence restored the influence of circMAPK9 deficiency on cell progression and inflammation in RA-FLSs. Therefore, these findings confirmed that circMAPK9 could modulate the aggressive phenotype of RA-FLSs by sponging miR-140-3p.

The circRNA/miRNA/mRNA network has been identified in diversiform diseases, such as hepatocellular carcinoma [32], gastric cancer [33], and systemic lupus erythematosus [34]. To explore the downstream mRNAs of circMAPK9/miR-140-3p network in RA, the possible targets of miR-140-3p were sought. Through identification, PPM1A was sponged by miR-140-3p. Philippe et al. attested that miR-19a/b could act as negative regulators in RA-FLSs by controlling TLR2 expression [35]. Nevertheless, whether miR-140-3p could regulate PPM1A level to affect RA progression is still ill-defined. In different cancers, PPM1A has been evinced to serve as a tumor suppresser or promoter [36, 37]. However, the precise function of PPM1A in RA progression has not been expounded. Lee et al. has disclosed that PPM1A was highly expressed in RA, and PPM1A expression was positively correlated with pro-inflammatory cytokine TNF level in RA synovial fluid [17]. In this study, the data showed that PPM1A enrichment was elevated in synovial tissue from RA patients and RA-FLSs, manifesting that PPM1A might be involved in RA progression. Interestingly, the rescue experiments indicated that PPM1A overexpression could abolish the impacts of miR-140-3p introduction on cell progression and inflammation in RA-FLSs, hinting that miR-140-3p could regulate the malignant development of RA via targeting PPM1A, which was parallel with the previous report [35]. Moreover, circMAPK9 was attested to positively regulate PPM1A expression by the crosstalk of miR-140-3p. Collectively, these data indicated that circMAPK9 might promote RA progression by regulating miR-140-3p/PPM1A axis.

This research conducted the in vitro experiments using the primary RA-FLSs, which represented the physiological function of RA patients. Furthermore, the involvement of circMAPK9/miR-140-3p/PPM1A network in RA-FLSs dysfunction was firstly confirmed, implying the significance and clinical expectation of this axis in RA advancement and therapy. Nevertheless, some limitations were still subsistent in the current study. For example, a larger number of RA patients and animal studies are needed in a further study in consideration of the limited number of patients and the restriction of in vitro experiments in this study. Besides, nanotechnology plays significant role in the area of bone-related therapy through providing attractive carrier options for delivery of therapeutic agents [38, 39]. The progressive damage of articular bone and cartilage was developed in RA patients, which might cause disability over time [40]. Recent evidence has suggested that bioengineered composite scaffolds and magnetic nanoparticles are effective promising therapeutic tools for RA remedy [41, 42]. Therefore, the combination of nanotechnology and molecular targeted drugs might be a most effective method for RA treatment.

## Conclusion

In conclusion, circMAPK9 interference constrained RA-FLSs proliferation, migration, invasion, inflammatory response, and expedited apoptosis possibly by enhancing miR-140-3p and lessening PPM1A expression. Our study first elucidated the circMAPK9/miR-140-3p/PPM1A regulatory network in RA-FLSs, offering a new perception about RA-FLSs progression, and offering a novel possible target for RA therapy.

## Data Availability

The data that support the findings of this study are available from the corresponding author upon reasonable request.

## Abbreviations

RA: Rheumatoid arthritis
FLSs: Fibroblast-like synoviocytes
PPM1A: Protein phosphatase magnesium-dependent 1A
ELISA: Enzyme-linked immunosorbent assay
circMAPK9: CircRNA mitogen-activated protein kinase
PBMCs: Peripheral blood mononuclear cells
FBS: Fetal bovine serum
qRT-PCR: Quantitative real-time polymerase chain reaction
siRNA: Small interfering RNA
DMSO: Dimethyl sulfoxide
FITC: Fluorescein isothiocyanate
WT: Wild-type
SD: Standard deviation
